# Assessing the responses of *Sphagnum* micro-eukaryotes to climate changes using high throughput sequencing

**DOI:** 10.7717/peerj.9821

**Published:** 2020-09-18

**Authors:** Monika K. Reczuga, Christophe Victor William Seppey, Matthieu Mulot, Vincent E.J. Jassey, Alexandre Buttler, Sandra Słowińska, Michał Słowiński, Enrique Lara, Mariusz Lamentowicz, Edward A.D. Mitchell

**Affiliations:** 1 Climate Change Ecology Research Unit, Faculty of Geographical and Geological Sciences, Adam Mickiewicz University, Poznań, Poland; 2 Laboratory of Soil Biodiversity, University of Neuchâtel, Neuchâtel, Switzerland; 3 Institute of Environmental Biology, Faculty of Biology, Adam Mickiewicz University, Poznań, Poland; 4 Department of Arctic and Marine Biology, Faculty of Biosciences Fisheries and Economics, University of Tromsø, Tromsø, Norway; 5 Laboratoire Ecologie Fonctionelle et Environnement, Université de Toulouse, CNRS, Toulouse Cedex, France; 6 Ecological Systems Laboratory, School of Architecture, Civil and Environmental Engineering, École Polytechnique Fédérale de Lausanne, Lausanne, Switzerland; 7 Swiss Federal Institute for Forest, Snow and Landscape Research, Site Lausanne, Switzerland; 8 Department of Geoecology and Climatology, Institute of Geography and Spatial Organization, Polish Academy of Sciences, Warsaw, Poland; 9 Past Landscape Dynamics Laboratory, Institute of Geography and Spatial Organization, Polish Academy of Sciences, Warsaw, Poland; 10 Real Jardín Botánico, Consejo Superior de Investigaciones Científicas, Madrid, Spain; 11 Jardin Botanique de Neuchâtel, Neuchâtel, Switzerland

**Keywords:** Wetlands, Protists, Warming, Water table manipulation, Food-web, Biodiversity, Community structure

## Abstract

Current projections suggest that climate warming will be accompanied by more frequent and severe drought events. Peatlands store ca. one third of the world’s soil organic carbon. Warming and drought may cause peatlands to become carbon sources through stimulation of microbial activity increasing ecosystem respiration, with positive feedback effect on global warming. Micro-eukaryotes play a key role in the carbon cycle through food web interactions and therefore, alterations in their community structure and diversity may affect ecosystem functioning and could reflect these changes. We assessed the diversity and community composition of *Sphagnum*-associated eukaryotic microorganisms inhabiting peatlands and their response to experimental drought and warming using high throughput sequencing of environmental DNA. Under drier conditions, micro-eukaryotic diversity decreased, the relative abundance of autotrophs increased and that of osmotrophs (including Fungi and Peronosporomycetes) decreased. Furthermore, we identified climate change indicators that could be used as early indicators of change in peatland microbial communities and ecosystem functioning. The changes we observed indicate a shift towards a more “terrestrial” community in response to drought, in line with observed changes in the functioning of the ecosystem.

## Introduction

Although much remains to be done before we can fully predict future climate variability (Ljungqvist et al., 2016), current projections suggest that climate warming will be accompanied by more frequent and severe drought events (Dai, 2013) thus affecting terrestrial communities. Amongst these, eukaryotic micro-organisms play key roles in ecosystem functioning as primary producers, decomposers predators and parasites (e.g., Fell et al., 2006; Geisen et al., 2018; Jassey et al., 2013a). Alterations in micro-eukaryotic community structure and diversity caused by ongoing environmental changes, including global warming and drought, may affect ecosystem functioning (Arndt & Monsonís Nomdedeu, 2016; Petchey et al., 1999), making it all the more necessary to include micro-eukaryotes in climate change studies (Cavicchioli et al. (2019).

Peatlands store one third of the world’s soil organic carbon (i.e., 500 ±100 GtC; Yu, 2012), but are particularly sensitive to climate induced perturbations, threatening their role in the C-cycle. Indeed, warming accompanied by drought which stimulates microbial activity (Bardgett, Freeman & Ostle, 2008; Freixa et al., 2017) leads to disruption of the peatlands’ function as C sinks and turns these ecosystems into carbon sources through increased respiration (Dorrepaal et al., 2009), with positive feedback effect on warming (Baird et al., 2013; Davidson & Janssens, 2006; Gorham, 1991; Yu, 2012). Peatland carbon balance is controlled by primary production (mainly by plants, to a lesser extent by autotrophic and mixotrophic micro-organisms; Jassey et al. (2015)) and decomposition (mainly by bacteria and fungi). Microbial activity is increased by micro-eukaryotic grazing on bacteria and fungi (e.g., Hahn & Höfle, 2001), implying that the standing biomass of bacteria and fungi does not necessarily correlate to decomposition intensity and their turnover may be enhanced under warmer or drier conditions. Therefore, identifying the factors that influence micro-eukaryotic community structure and diversity is crucial for our understanding of potential peatland feedbacks on climate warming.

The significance of peatlands in the global C cycle is in large part due to the unique biological characteristics of *Sphagnum* mosses, the main builders of high latitude peatlands. *Sphagnum* mosses are adapted to acidic, water-logged and nutrient-poor conditions, which they contribute to create (Rydin, Jeglum & Hooijer, 2006). Clymo & Hayward (1982) suggested that more C might be stored in *Sphagnum* (living and dead) than in any other plant genus. *Sphagnum* and its associated specific microbial community constitutes a microecosystem called “sphagnosphere” and this complex system may be the key to the nutrient cycling in peatlands (Chiapusio et al., 2013), and hence to its C-sequestrating function. *Sphagnum*-associated microbial communities change in composition and also functionally along climatic (Singer et al., 2019) and micro-environmental gradients (Mitchell et al., 2003). The *Sphagnum* microbiome may supports its host through nutrient supply and defence against pathogens (Bragina et al., 2013; Raghoebarsing et al., 2005), and ultimately the ecosystem under changing climate (Bragina et al., 2014).

Micro-eukaryotes include protists, fungi, microscopic animals and eukaryotic “micro-algae”. These organisms are essential components of peatland ecosystems (Lara et al., 2011; Reczuga et al., 2018). Phototrophic micro-eukaryotes (=“microalgae”, here excluding cyanobacteria) in peatlands play key roles in photosynthetic C fixation and thus in primary production (e.g., Jassey et al., 2015; Schmidt, Dyckmans & Schrader, 2016), while organic matter cycling is driven by phagotrophic protists predating on bacteria and fungi (Jürgens & Sala, 2000; Ribblett, Palmer & Wayne Coats, 2005). Micro-eukaryotes take part in nutrient cycling by transferring nutrients to higher trophic levels through microbial food web (Crotty et al., 2012; Gilbert et al., 1998). For example, soil protists grazing on bacteria and other microorganisms release nitrogen and other nutrients making them available for plants (Bonkowski, 2004). In *Sphagnum*-associated microbial community, large amoebae are top microbial predators and consume a wide variety of prey, such as bacteria, fungi, micro-algae and micro-invertebrates (Jassey et al., 2012). Micro-eukaryotes can also act as parasites of other organisms (Geisen, 2016b; Geisen et al., 2015) and play numerous important, previously unrecognized, functions (Geisen, 2016a). Despite growing knowledge about the diversity of eukaryotic microbes associated with *Sphagnum* (Jassey et al., 2013a; Jassey et al., 2015; Singer et al., 2019; Tsyganov et al., 2012), still little is known about their response to climate changes.

Warming has indirect effects on microbial food web structure, altering the community composition and ecosystem functioning (Jassey et al., 2011). For instance, Tsyganov et al. (2012) have shown that the response to climate change differs among seasons, and that testate amoebae, a dominant group of microbial top predators in peatlands (Gilbert et al., 1998; Jassey et al., 2013b; Jassey et al., 2012; Meyer et al., 2013; Mieczan, 2007) are especially sensitive during the growing season. Tveit et al. (2015) showed that warming caused an increase in the relative abundance of Cercozoans (phagotrophic protists), higher substrate turnover and microbial activity as well as changes in methanogenic pathways. Jassey et al. (2013a) demonstrated that warming reduced the abundance of top predators while the biomass of bacteria increased. Such changes in microbial community structure can potentially alter the above- belowground linkages, destabilizing the C cycle (Jassey et al., 2013a).

Peatlands are particularly sensitive to drought, as these ecosystems are primarily controlled by the high water level that creates anoxic conditions allowing peat to accumulate. Indeed, severe drought might induce changes in peatland ecosystem function, turning them into carbon sources (Jassey et al., 2018). Drought alters community composition (Potter et al., 2017), shifting the community towards species adapted to dry habitats (Koenig, Mulot & Mitchell, 2018). Prolonged drought conditions might lead to loss of key species or decrease their abundance below the level necessary to drive ecosystem function (Jenkins & Boulton, 2007). The resulting destabilization of the food-web structure might then cross a tipping point and shift the C balance (Dakos et al., 2019; Lamentowicz et al., 2019).

The goal of this study was to assess the diversity and community composition of eukaryotic microorganisms inhabiting *Sphagnum* in peatlands, with a main focus on protist communities, and their response to experimental drought and warming. We hypothesized that (1) the diversity and community structure of micro-eukaryotes vary in response to drought and warming. More specifically, we hypothesised that (2) drought causes a decrease in the relative abundance and diversity of primary producers, as microalgae predominantly inhabit aquatic environments (e.g., Ray et al., 2019), (3) an increase in the diversity and relative proportion of fungi, as it has been suggested that drought leads to increased dominance of fungi (Jensen et al., 2003), and this agrees with observational evidence for a higher contribution of fungi to overall microbial biomass under drier conditions (Mitchell et al., 2003), although responses might be site-specific (Jaatinen et al., 2007); (4) the effects of drought are exacerbated by the effects of warming; and (5) drought has a stronger effect than warming. To this aim, we conducted a passive warming and water table depth field manipulative experiment and assessed the diversity of micro-eukaryotes by high throughput sequencing (Illumina HiSeq) of the SSU rRNA V9 region.

## Materials and Methods

### Study site and field experiment

The study site, Linje peatland (53°11′15″N, 18°18′34″E), is situated in northern Poland within the Complex of Chełmno and Vistula Landscape Parks at 91 m a.s.l. The climate is classified as warm temperate, fully humid with warm summers (Kottek et al., 2006) or cold without dry season with warm summer (Beck et al., 2018). Average annual precipitation in this region is between 500–550 mm and average annual air temperature is between 7.5–8.0 °C. The peatland is dominated by *Sphagnum fallax*, *Eriophorum vaginatum*, *Oxycoccus palustris*, *Andromeda polifolia*, *Aulacomnium palustre*, and *Drosera rotundifolia* (Boiński & Boińska, 2004). The experimental site included also shrubs and young trees such as *Betula nana* and *Pinus sylvestris,* respectively.

**Figure 1:**
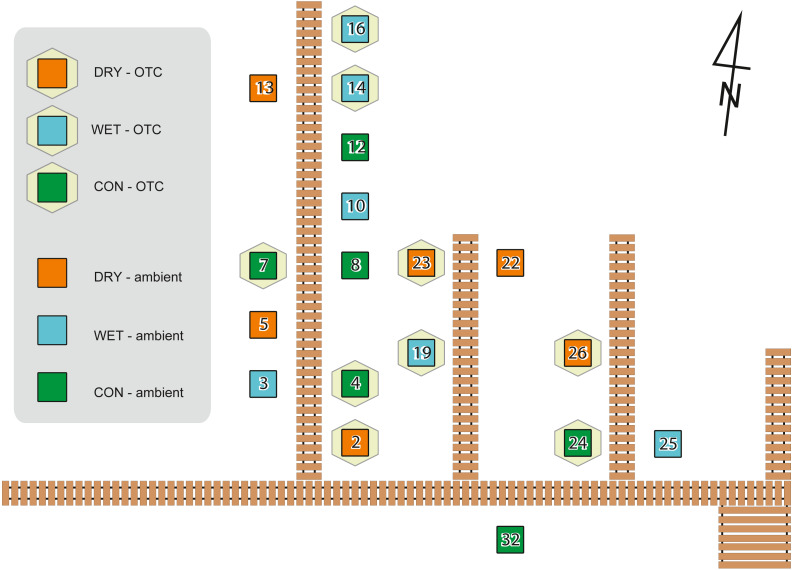
Experiment design showing localization of the plots on the experimental site with its board walk. Hexagons indicate warming treatment (OTC), squares indicate plots without OTC. Water table treatments are indicated with colours as shown in the legend. Adapted from Lamentowicz et al. (2016).

To simulate warming, we used Open Top Chambers (OTCs), which are commonly used in manipulative experiments *in situ* (e.g., Delarue et al., 2011; Jassey et al., 2013a; Marion et al., 1997), while water table depth was manipulated by adding or removing peat (Lamentowicz et al., 2016; Reczuga et al., 2018). The advantage of manipulative field experiments is that they are performed under realistic environmental conditions with community composition and abiotic drivers as they appear and work in the field (Underwood, 2009). Field experiments were approved by The Regional Directorate for Environmental Protection in Bydgoszcz (WPN.6205.70.2011.KLD). The experimental design with plots and treatments are given in Fig. 1.

In August 2012, experimental plots were defined according to a full factorial design involving two temperatures and three water table depths. The temperature increase effect was manipulated using OTCs or let under ambient temperature as control (AMB). OTCs are passive warming systems that allow increasing mean temperature by ca. 1.2 °C to 1.8 °C (Marion et al., 1997). The hexagonal OTC chambers were constructed with transparent polycarbonate material. The internal diameter between opposite corners was 250 cm at the base. To allow for air circulation, the OTCs were placed 10 cm above the soil surface. We manipulated water table depth by adding or removing 10 cm layers of peat in 1-m^2^ plots. For each plot four subplots (50 × 50 cm and 30 cm thick) were cut. They were attributed to different measures (e.g., moss sampling for this study, respiration, biomass). Each plot belonged to one of three water table treatments: control (subplots cut and put back in place, abbreviation: CON), wet (10 cm of peat removed beneath each subplot before putting the subplot back in place, abbr.: WET), dry (10 cm of peat added from the wet treatment before putting the subplot back in place, abbr.: DRY). CON treatment was cut and put back in place to control for the disturbance introduced by cutting. Each plot was a combination of one of the three water table treatments and one of the two warming treatments. All six combinations were replicated three times, giving a total of 18 plots (subplots within the experimental plot belonged to the same treatment). Treatments were assigned to the plots randomly. Air temperature was recorded in each plot at 30 cm height using HOBO U23 Pro v2 data loggers (Onset Computer Corporation, USA) and the water table depth (WTD) was measured at each sampling (given in cm below the soil surface). Whenever we are referring to the measured values we use abbreviation WTD, while term “water table treatments” is used when we refer to the levels of manipulation (i.e., DRY, CON, and WET). A subset of the data from the experiment described in this study has already been published elsewhere (Jassey et al., 2018; Lamentowicz et al., 2016; Samson et al., 2018). The experimental design was described by Lamentowicz et al. (2016), where further details can be found. The effect of warming manipulation on temperature has been published in Samson et al. (2018), a study describing influence of warming and water table treatments on carbon dioxide release.

### DNA extraction, PCR amplification and next-generation sequencing

Two to three individual *Sphagnum* stems (top three cm, including capitula) were sampled in three different seasons in each of the plots on May 16th (spring), August 20th (summer) and November 7th (autumn) in year 2013 and fixed in LifeGuard^™^ Soil Preservation Solution (MoBio, Carlsbad, CA USA). The plots were placed in monospecific *Sphagnum fallax* lawns and therefore we did not consider the species level identification of the sampled *Sphagnum*. In total 54 samples were taken (6 combinations of treatments * 3 replicates * 3 seasons). We sampled only the top part of the mosses as previous studies proved this part to be the most sensitive to the changes in the water table depth and warming manipulations (Basińska et al., 2020; Reczuga et al., 2018). DNA was extracted using the PowerSoil®DNA isolation kit (MoBio, Carlsbad, CA USA) according to the manufacturer’s instruction. The DNA was extracted from two to three sampled *Sphagnum* mosses, which is approximately 0.3 –0.5 g of material per plot. PCR amplification of the SSU rDNA V9 region was carried out with eukaryotic-specific primers 1380F (CCCTGCCHTTTGTACACAC) and 1510R (CCTTCYGCAGGTTCACCTAC) (Amaral-Zettler et al., 2009). PCR reactions were conducted according to the following conditions: denaturation at 95 °C for 3 min, 25 cycles at 94 °C for 30 s, 57 °C for 30 s and 72 °C for 60 s and final extension at 72 °C for 10 min (Amaral-Zettler et al., 2009). PCR reactions were run with a SensoQuest Labcycler (GmbH, Göttingen, Germany) with 3 l of environmental DNA extract, 4 μL of 5X Colorless GoTaq®Buffer, 0.6 μl of each primer, 0.6 μl of dNTP Mix 10 mM (Promega, Dübendorf, Switzerland) and 0.2 μl of 0.05 U/ μl Go Taq Polymerase (Promega, Dübendorf, Switzerland). Each PCR reaction was performed with a positive and a negative control. Negative controls included PCR reaction mix with DNA extract replaced with water. The purification step was done using the Wizard®SV Gel and PCR Clean-Up System (Promega, Madison, WI USA). Library preparation and paired end sequencing was performed at the iGE3 Genomics Platform of the University of Geneva, Geneva, Switzerland using Illumina Hiseq technology (2 × 150 bp).

### Sequence analysis

The computational pipeline used to analyse the reads includes the following steps: trimming of the tagged primer sequences, quality check, removal of rare sequences (i.e., OTU’s occurring less than 3 times in the full dataset), clustering, and taxonomic assignation. For a given read, the quality check was based on moving windows of 50 nucleotides. The probability of incorrect base call was calculated for every nucleotide based on the phred score and arithmetic mean of these probabilities was calculated for every window. To avoid artefactual sequences; for the same purpose, we kept only reads that were present at least three times in the full dataset (De Vargas et al., 2015). Sequence clustering was then performed using Swarm v. 1.2.12 (Mahé et al., 2014). Taxonomic assignation of the resulting OTUs (Operational Taxonomic Units) was done by pairwise alignment of the dominant sequences of each OTU against a selection of dereplicated V9 regions from the PR^2^ database (Guillou et al., 2013) using ggsearch36 v. 36.3.6 (Pearson, 1999). The sequences assigned to Metazoa and Embryophyceae were excluded from further analysis. In order to remove possible false positive sequences only OTU’s occurring at least 10 times in the dataset were retained for further numerical analyses. Based on the taxonomic assignation, each OTU was assigned to a functional group (autotrophs, mixotrophs, parasites, osmotrophs and phagotrophs) according to Table 1. The assignation of OTUs to the functional groups was based on the expert curation (Enrique Lara). OTUs annotated in PR^2^ database with an unknown taxonomic identity (Guillou et al., 2013), were cross-checked individually by aligning them against the NCBI’s nucleotide database using BLAST algorithm with default parameters.

**Table 1:** Taxonomic groups corresponding to each functional group. The list of taxa is based on OTU assignations using the PR2 database.

Functional group	Taxonomic groups from PR ^2^ database assigned to the given functional group
Autotrophs	Dinophyceae, Chlorophyceae, Mamiellophyceae, Trebouxiophyceae, Klebsormidiophyceae, Zygnemophyceae, Bacillariophyta, Eustigmatophyceae
Mixotrophs	Euglenozoa, Cryptophyceae, Prymnesiophyceae, Chrysophyta (pro parte)
Phagotrophs	Colpodea, Heterotrichea, Litostomatea, Nassophorea, Oligohymenophorea, Phyllopharyngea, Prostomatea, Spirotrichea, Mycetozoa-Myxogastrea, Variosea, Discosea-Longamoebia, Heterolobosea, Centroheliozoa, Katablepharidaceae, Choanoflagellatea, Nucleariidea, Endomyxa, Filosa-Granofilosea, Filosa-Imbricatea, Filosa-Metromonadea, Filosa-Sarcomonadea, Filosa-Thecofilosea, Bicoecea, Hyphochytriomyceta, Labyrinthulea, MAST, Chrysophyta (pro parte)
Osmotrophs	Ascomycota, Basidiomycota, Blastocladiomycota, Zoopagomycota, Mucoromycota
Parasites	Apicomplexa, Cryptomycota, Rhyzophidiales, Ichthyosporea, Oomycota (Peronosporomycetes)

### Growing degree day

To link the micro-eukaryotic communities with warming, we used a growing-degree day (GDD) approach, also known as accumulated degree day (ADD). The GDD is widely being used to predict plant and insect phenology and has more recently been used to relate e.g., grasshopper and butterfly community changes to climate change (e.g., Cayton et al., 2015; Nufio et al., 2010).

\begin{eqnarray*}\mathrm{GDD}=  \frac{{T}_{\mathrm{max}}+ {T}_{\mathrm{min}}}{2} - {T}_{\mathrm{base}} \end{eqnarray*}

where,

T_max_ are daily maximum air temperature in ^∘^C,

T_min_ are daily minimum air temperature in ^∘^C,

T_base_ is a base temperature, equal to 10 °C.

For each sample, the GDD was calculated for 135 days—the number of days since the beginning of the year until the first sampling.

### Numerical analysis

All statistical analyses were performed in R version 3.5.1 (R Core Team, 2012) using RStudio Version 1.1.423 (RStudio Team, 2012). Whenever measured water table depth is used as a variable, we call it WTD, while whenever term “water table treatments” is used we refer to the levels of manipulation (i.e., DRY, CON, WET). The warming treatment effect on GDD and water table treatment on WTD was tested using ANOVA followed by Tukey multiple comparisons of means. We tested for treatment effects (both warming and water table manipulation) on the diversity patterns of micro-eukaryotic communities using the Shannon diversity index, rarefied OTU richness and evenness. OTU richness was estimated using *rarefy* function in the *vegan* package (Oksanen et al., 2014) at the sample size of 2410 (minimum number of reads across all samples) to compensate for the variability of the sample sizes. The sequencing effort between the samples was compared by drawing rarefaction curves using *rarecurve* function and by comparing slope calculated using *rareslope* function of the *vegan* R package (Oksanen et al., 2014). The slope was also calculated at the sample size of 2410. To test the significance of differences along measured WTD and temperature gradient (calculated GDD) linear mixed effects models with measured WTD and calculated GDD as fixed factors and season nested in plot as a random effect to account for the differences between plots across time. The assumptions of homoscedasticity and normality were previously tested. The tests were performed using *lme* function in the nlme package (Pinheiro et al., 2019). To further test the correlation between diversity and WTD and GDD, we calculated Pearson’s correlation using *cor.test* function.

Subsequently, to test the response of micro-eukaryotes to the GDD, measured water table depth (WTD (cm)) and seasons, at the community level, we performed an RDA (Redundancy Analysis) based on Hellinger-transformed OTU counts. The RDA was done using *rda* function in the *vegan* package (Oksanen et al., 2014). Significance tests of each variable and axis in RDA models were tested using permutation tests (*anova.cca* function in the *vegan* package). To identify micro-eukaryotic indicators of climate change, we used Dufrene-Legendre indicator species analysis (Dufrêne & Legendre, 1997) and *multipatt* function in the *indicspecies* package (De Cáceres & Legendre, 2009). To manually verify taxonomic assignation against more wholesome database, the selected indicators’ (with the highest indicator value) sequences were cross-checked by aligning them against the NCBI’s nucleotide database using BLAST algorithm with default parameters.

## Results

### Warming and water table manipulation effects on microclimatic conditions

Mean maximum daily temperature was about 1.1–1.2 °C higher in the OTC than in the ambient plots (Samson et al., 2018). The warming effect varied seasonally and over daytime, reaching a maximum effect of Δ 4.1 °C on 5th of May 2013 and causing a slight cooling effect during winter and during the night (Lamentowicz et al., 2016). Mean daily air temperature was calculated on the basis of 10-minute averages. For each sampling, the temperature of the month prior to the sampling was calculated as the average of the mean daily air temperature. This mean was higher in the OTC than in ambient plots in spring and summer periods (by 0.54° and 0.43°, respectively). In each season WTD was higher (i.e., lower water table) in DRY as compared to the CON and WET. Mean WTD in DRY was 15 cm, 46 cm and 25 cm in spring, summer and autumn sampling, respectively, while in CON it was nine cm, 40 cm and 19 cm and in WET it was eight cm, 40 cm, 19 cm (Table S1, Fig. S1). Significant differences were observed between CON and DRY as well as between WET and DRY (Tukey, *p* < 0.05), while no significant difference was observed between WET and CON (Tukey, *p* = 0.6). GDD differed significantly (ANOVA, p <0.05) between AMB and OTC across seasons, with mean GDD of 76, 732 and 549 in AMB spring, summer and autumn periods, respectively, while OTC mean GDD was 101, 847 and 615 in spring, summer and autumn periods, respectively (Table S2). Significant differences between GDD in AMB and OTC were observed at each season (Tukey, *p* < 0.05). The environmental variables in experimental plots are provided in Data S1.

### Micro-eukaryotic community structure

The Illumina sequencing generated 37,804,202 raw eukaryotic SSU V9 reads. Of these, the number dropped to 29,791,631 after quality check. These sequences were then clustered in 2,043 OTUs, reduced to 1,397 OTUs after removing sequences assigned to Metazoa or Embryophyceae. The abundance of each of the 2,043 OTUs in experimental plots is shown in Data S2, while the dominant sequence of each OTU is shown in Data S3. For ecological analysis, all assignations with similarity below 80% were excluded, leaving 1,226 OTUs. The majority of OTUs were assigned to the following five eukaryotic supergroups in decreasing order of taxonomic richness (i.e., 614, 214, 152, 99 and 83 OTUs): Opisthokonta, Alveolata, Archaeplastida, Rhizaria, respectively. A total of 369 OTUs (26.4% of the total number of OTUs) were assigned to taxa with a similarity above 97%. Among these, 250 were assigned to fungi and 119 to other taxa. Fungi OTUs reaching 97% of identity with the database constituted 40% of all fungi while only 16% of the other taxa reached the identity threshold illustrating the knowledge gap between fungi and protists.

### Warming and water table manipulation effects on micro-eukaryotic diversity

**Figure 2:**
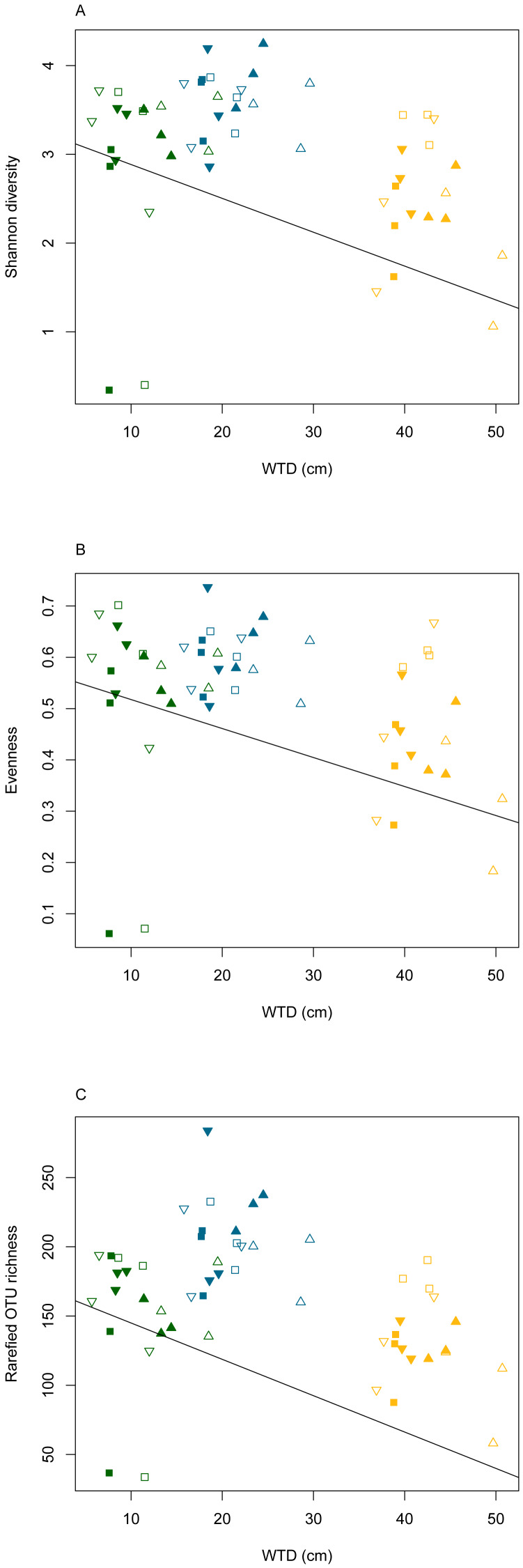
Diversity measures (Shannon diversity - A, evenness - B and rarefied OTU richness - C) plotted against water table depth (WTD (cm)). Colours indicate different seasons: green for spring, yellow for summer and blue for autumn sampling campaigns. Filled symbols indicate warming (OTC), empty symbols indicate ambient temperature. Water table treatments are indicated by symbols: ‘■’ = CON (control); ▴ = DRY and ‘▾’ = WET. The solid line represents the coefficients estimated in the linear mixed effects model.

The rarefaction curves (Fig. S2) clearly showed that the sequencing effort was not saturated at the sample level. However, when considering the whole dataset, the rarefaction curve indicated that the majority of micro-eukaryotic diversity was recovered. To compensate for the saturation variability among treatments, the OTU richness was calculated on a rarefied community in order to compare the effect of the different treatments. We did not observe significant differences in the slope of the rarefaction curves among water table or warming treatments (ANOVA, *p* = 0.6 and *p* = 0.9, for warming and water table treatments, respectively; Fig. S3). Linear mixed effect models revealed that Shannon diversity, evenness and rarefied OTU richness differed significantly along WTD and GDD (ANOVA, *p* < 0.01, Table S3). All diversity indices declined significantly with increasing WTD (Fig. 2, cor = −0.3 for all diversity measures, *p* < 0.05). No significant correlation was observed between GDD and biodiversity metrics. Shannon diversity and OTU richness were higher in autumn as compared to summer and spring. No significant difference was observed between warming treatments. The Shannon diversity, evenness, OTU richness and rarefied OTU richness in each analysed sample are given in the Table S4.

### Micro-eukaryotic community response to the experimental manipulation

**Figure 3:**
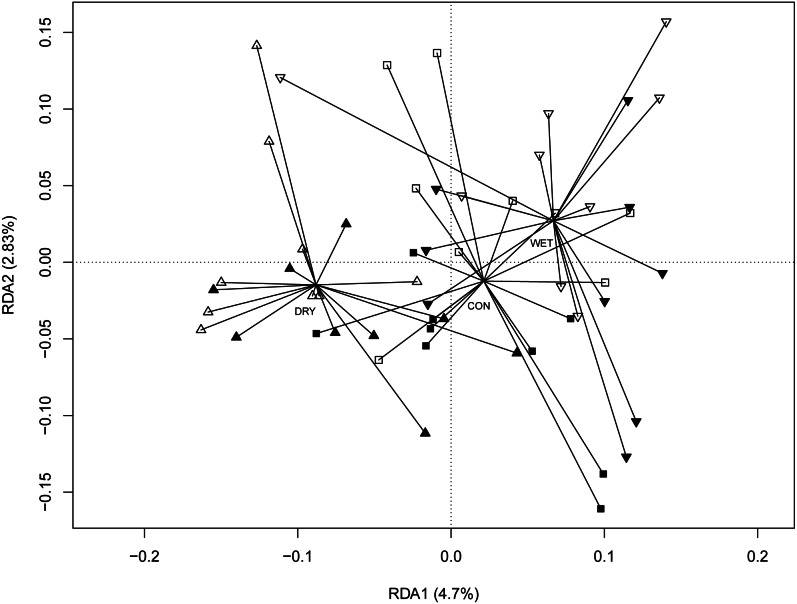
Redundancy analysis (RDA) ordination diagram showing patterns of eukaryotic communities on *Sphagnum* shoots as assessed by OTUs in the different plots. Season (spring, summer and autumn sampling campaigns) is specified as a conditioning variable (“covariable”) partialled out before analysis. Filled symbols indicate warming (OTC) and empty symbols indicate ambient temperature. Water table treatments are indicated by symbols: ‘■’ = CON (control); ‘▴’ = DRY and ‘▾’ = WET. Centroid of each water table treatment is connected with its members with lines. Percentages of variance explained are given for each axis.

The redundancy analysis (RDA) revealed significant correlations between micro-eukaryotic communities and WTD (*anova.cca* permutation test, *p* = 0.001), GDD (*p* = 0.001) and season (*p* = 0.001). The full RDA model explained 22.5%, most of this (16.6%) being explained by seasons. To disentangle the seasonal effects from the influence of other environmental factors, we performed RDA with season as a conditional variable (Fig. 3). Here again, WTD (*p* = 0.014) and GDD (*p* = 0.017) were significant. The partial RDA model explained 5.9% (*p* = 0.003) of the variance. RDA model with water level and temperature treatments as variables and with season as a conditional variable revealed significant differences between water level treatments (*p* = 0.001), while the difference between temperature treatments was insignificant (*p* = 0.147; Fig. S4).

**Figure 4:**
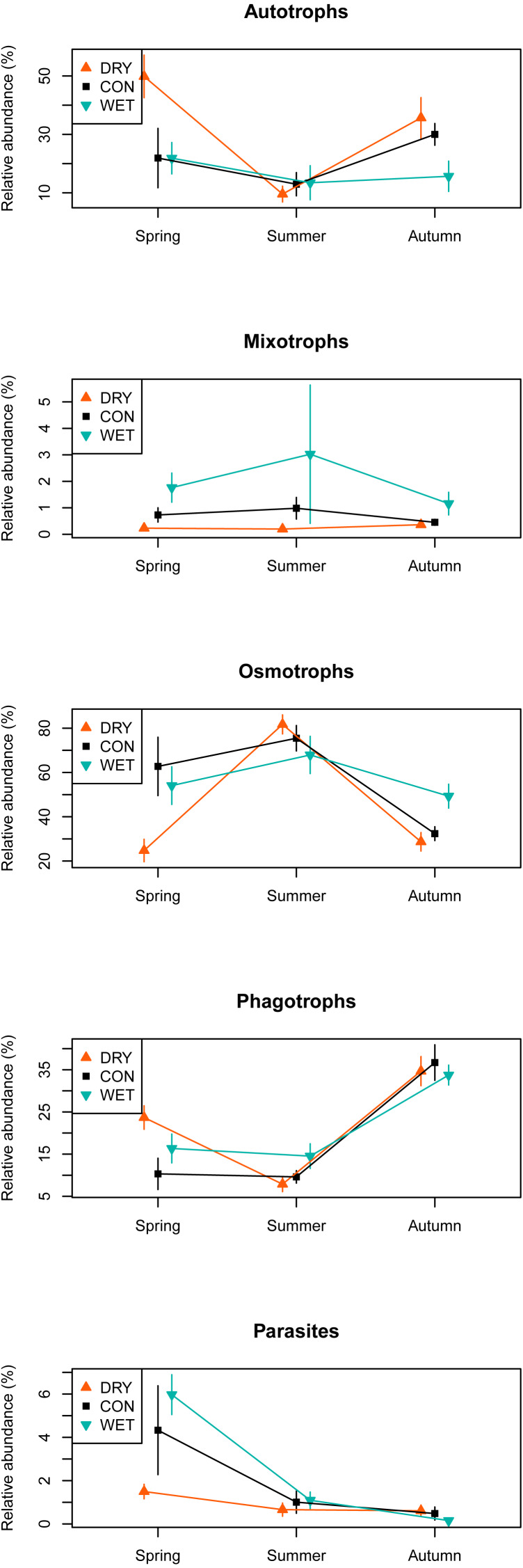
Relative abundance of sequences assigned to functional groups by water table treatment and by season in the different plots. Taxonomic groups corresponding to each functional group are given in Table 1. Bars indicate standard errors. The relative abundance (%) is a number of OTUs in a given functional group, divided by the total number of all OTUs combined.

Among the 1,226 OTUs, 216 were considered autotrophs, 52 mixotrophs, 42 parasites, 579 osmotrophs and 319 phagotrophs (Table 1). Eighteen OTUs that could not be placed phylogenetically within any phylum, and therefore could not be functionally assigned, were excluded from the analysis. In the DRY treatment in spring, we observed a higher relative abundance of autotrophs and phagotrophs and a lower relative abundance of osmotrophs and parasites as compared to the WET and CON treatments. No such difference between treatments was observed in summer and autumn (Fig. 4).

### Micro-eukaryotic indicators of global change

**Figure 5:**
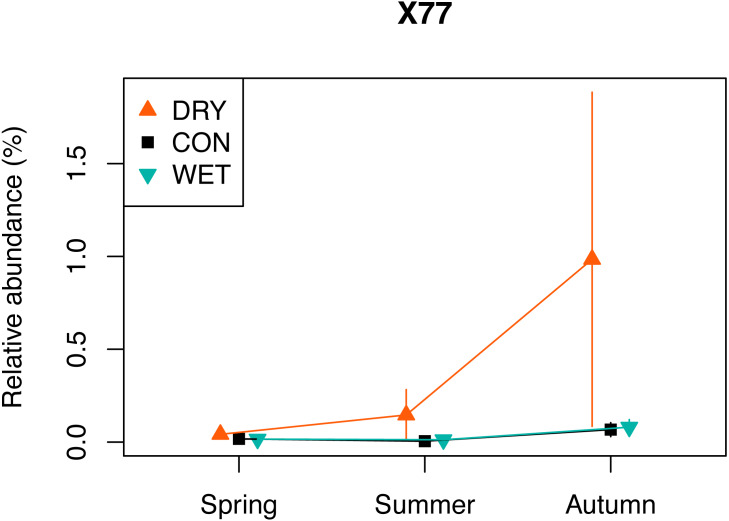
Relative abundance of a selected DRY indicator (OTU X77) with the highest indicator value (0.96), assigned to Fungi, *Cortinarius* (see also Table 2). The relative abundance (%) is a number of a given OTU, divided by the total number of all OTUs combined.

The indicator species analysis with each treatment as a separate group, allowed identifying 109 indicator OTUs (Table S5). A total of 96 OTUs were DRY indicators, 10 were WET indicators and three were identified as CON indicators (*p* < 0.05). Among the 96 DRY indicators, 28 were assigned to Fungi, 23 to Chlorophyta, 17 to Ciliophora, 14 to Cercozoa, six to Dinophyta, two to Cryptophyta and Stramenopiles, one to Apicomplexa, Discoba, Mesomycetozoa and Streptophyta. The DRY indicator with the highest indicator value was OTU_X77 (100% similarity with *Cortinarius* sp.) which had an indicator value of 0.963. The relative abundance of this OTU was highest in autumn (Fig. 5). Among the 10 DRY indicators with the highest indicator value, five were assigned to Archaeplastida (including four assigned to Trebouxiophyceae), four to Fungi and one to Alveolata (Colpodidae; Table 2). The increase of the relative abundance of autotrophs in spring corresponded to indicator algae adapted to dry soil environment and capable of symbiosis with lichens, such as *Elliptochloris*, (Trebouxiophyceae, Metz et al., 2019). The relative abundance of OTUs assigned to *Elliptochloris*, predominantly terrestrial taxa (Gustavs et al., 2017; Rindi, Hodkinson & Jones, 2011), increased from 1% in CON plots in May to 6% in DRY plots in May (Fig. S5), showing a shift towards a more terrestrial community.

A total of 27 indicators were identified for ambient vs OTC, nine of which were indicators of ambient temperature and 18 were indicators of warming (*p* < 0.05; Table S6).

## Discussion

Micro-eukaryotes, as key components of terrestrial ecosystems functioning, are vulnerable to ongoing climate change, which threatens global biodiversity (Geisen, Wall & Putten, 2019; Urban, 2015) making it urgent to gain insight into upcoming changes. Micro-eukaryotes are vulnerable to extinctions (Cotterill et al., 2013) and species loss may lead to cascading effects (Pearse & Altermatt, 2013) and disrupt ecosystem functioning (Santschi et al., 2017). It is expected that in stable habitats where the majority of species are near their thermal optima, taxonomic diversity will decrease with warming (Woodward, Perkins & Brown, 2010). Analogically, in stable peatlands, where the majority of micro-eukaryotes are near their humidity optimum, the diversity in the *Sphagnum* layer can be expected to decrease under drier conditions. Here, we confirm these hypotheses as diversity was negatively correlated with WTD. The diversity decrease might result in local loss of specialized species adapted to permanently water-logged conditions. These disappearing species may be replaced by taxa better adapted to new conditions.

**Table 2:** Micro-eukaryote indicator OTUs and corresponding taxonomic assignation of DRY indicators with the highest indicative value. *P*-value indicate the probability to have a higher IndVal by chance.

Taxonomic assignation of OTUs		OTU code	Accepted taxonomic rank	Indicator value	*p*-value	Percent identity
Alveolata	Colpodida	X227	Colpodidae	0.887	0.001	95
Archaeplastida	Trebouxiophyceae	X104	*Elliptochloris reniformis*	0.941	0.001	100
	Microthamniales	X118	Environmental seq	0.933	0.001	100
	Trebouxiophyceae	X100	*Coccomyxa* sp.	0.916	0.001	100
	Trebouxiophyceae	X115	Environmental seq	0.864	0.002	100
	Trebouxiophyceae	X250	*Elliptochloris* sp.	0.84	0.002	95
Fungi	Agaricomycotina	X77	*Cortinarius* sp.	0.963	0.001	100
	Pezizomycotina	X43	*Venturia* sp.	0.957	0.019	100
	Agaricomycotina	X64	Agaricomycete	0.896	0.017	95
	Agaricomycotina	X180	*Lactarius* sp.	0.827	0.004	100

Conserving biodiversity is viewed as essential in part because our current knowledge does not allow to state which species are critical to the functioning of ecosystems and provide resilience and resistance to global changes (Chapin III et al., 2000). This is particularly true for micro-eukaryotes, whose significance in functioning of ecosystems has only recently been re-brought to light and which we know have important and sometimes unrecognized functions (Geisen et al., 2017).

The community composition of *Sphagnum*-associated micro-eukaryotes has only recently started being studied through molecular approaches. High throughput sequencing (HTS) studies revealed that taxa previously believed to appear almost only in aquatic environments are present in moss-associated communities. For example, Dinoflagellates were recently reported in moss-associated communities (Heger et al., 2018) and Chrysophyceae and Kinetoplastida in soils (Lentendu et al., 2018). We also recorded these three groups in our study. As we gain insight into the community composition of micro-eukaryotes, the information on the function of the micro-eukaryotes associated with *Sphagnum* remains largely unknown. Identifying which micro-eukaryotes form close associations with *Sphagnum* mosses and what influence they have on their host and on the functioning of the ecosystem, remain open questions.

Accordingly, the DRY plots were characterized by the presence of predominantly terrestrial taxa, among which many Trebouxiophyceae, such as *Elliptochloris* (Gustavs et al., 2017; Rindi, Hodkinson & Jones, 2011). Soil microalgae are better adapted to dehydration, rapid changes in temperature and intense solar radiation than their freshwater counterparts (Gray, Lewis & Cardon, 2007). Several members of the Trebouxiophyceae form symbiotic associations with lichenizing fungi (Metz et al., 2019). These associations are primarily terrestrial and most of them cannot survive permanently water-logged conditions (Hawksworth, 2000).

One of the indicators of drought (OTU_X77) is related to *Cortinarius sp.*, (Agaricomycetes). This ectomycorrhizal fungi, commonly found in peatlands, is associated with the roots of *Picea*, *Larix*, *Salix*, and *Betula* (Thormann, 2006). *Betula nana* occurred in our plots and *Pinus sylvestris* grew in proximity of the plots, and both of these species could thus be the host for the OTU_X77. Encroachment of shrub and tree species is observed in pristine and drained peatlands in response to water loss and/or warming (Berg et al., 2009; Fay & Lavoie, 2009). It determines a shift in plant formation towards forest ecosystem. This transition increases water losses through evapotranspiration (Fay & Lavoie, 2009). Our observation of the drought indicator OTU_X77 may, therefore, be an early sign of these transition. However, contrary to our expectation, we did not observe an overall increase in diversity and relative abundance of fungi.

The HTS approach allows for DNA-based species identification and biodiversity assessment (Taberlet et al., 2012) but does not directly inform on the species’ function, activity or metabolic state. While functions can, to some extent, be inferred from taxonomy (Adl et al., 2019), detailed knowledge about the function of many protists is still missing. Nevertheless, our study allows already to identify taxa which should be studied in more detail in priority to better understand how the observed changes in their abundance will affect ecosystem functioning.

The presence of these new taxa suggests a shift in the species composition and structure of microbial communities in response to water table drawdown towards terrestrial taxa. We also observed an increase in the diversity and relative abundance of terrestrial algae during the late spring, at the time of spring blooms (Fig. 4). Earlier algae blooms can be triggered by climate warming (Peeters et al., 2007) as warming might physiologically facilitate algal growth (Barton et al., 2003). Shifts in algal blooms timing are known to affect biological interactions within food-webs, such as competition (Peeters et al., 2007). Therefore, further studies are necessary to better understand by which mechanism drought stimulates the appearance of terrestrial algae and its consequences for the food-web. The drought simulated in this work, as a result of the manipulation, was probably more severe than the simple water table fall of about 10 cm. Possible cause of such scenario is a change in the peat position in boundary layer, and so altered evapotranspiration leading to more extreme drying. We can also expect more convective effects further enhancing the drought effect.

Changing biodiversity is affecting ecosystem functioning and the resilience of ecosystems to environmental change (Chapin III et al., 2000). For example, increased species richness may accelerate the decomposition of peat as a result of synergistic effects (Salonius, 1981). These effects are likely to be observed in species-poor ecosystems, where the occurrence of new species entails new functionalities (Vitousek & Hooper, 1994). In species-rich ecosystems, the impact of increased species richness on ecosystem processes is likely to be lowered due to functional redundancy (Chapin III et al., 2000; Johnson et al., 1996). Although there is no universal link between species diversity and ecosystem processes, certain processes are being fulfilled only by a handful of species and loss of these species might have huge consequences for ecosystem functioning (Chapin III et al., 2000; Kardol et al., 2016). Due to the scarcity of invertebrates in *Sphagnum*, micro-eukaryotic communities play major roles in the functioning of these ecosystems. Identifying *Sphagnum*-associated species which are vulnerable to climate change and inferring their function is useful to better predict future changes in the underlying peatland functioning and possible species loss (Midgley et al., 2002).

In a changing climate, the vulnerability of ecosystems is more likely to depend on community composition than on biodiversity (Chapin III et al., 2000). Changes in the relative abundance in favour of autotrophs at the expense of osmotrophs might affect the functioning of the ecosystem either by decreasing or by increasing decomposition rates, depending on how the food web structure responds. Increases in decomposition rates are, however, likely to occur deeper in the soil horizon were most of the decomposition processes take place, while our study investigated only the top part of the moss stem, where living *Sphagnum* dominates. In the DRY treatment, we identified some fungal indicators and we can expect that the appearance of these taxa might entail new functionalities in the ecosystem. Indeed, in the same experiment, (Jassey et al., 2018) did observe an increase in multifunctionality of enzymes in the DRY treatment. As pointed out by Kostka et al. (2016) *Sphagnum*-associated microbes might act as keystone species regulating C and N flow in peatlands. Changes in their community may therefore have consequences for the overall functioning of peatlands and their feedback on climate change.

## Conclusions

Our study revealed substantial changes in the diversity and community structure of micro-eukaryotes, both at the OTU and functional levels, in response to experimental manipulation of temperature and water table depth, confirming our first hypotheses. Regarding the responses to water table variation, we observed a decrease in abundance and diversity of micro-eukaryotes, an increase in the relative abundance and a decrease in the diversity of eukaryotic micro-algae along with the water table depth. The increase in the number of autotrophs was due to the occurrence of predominantly terrestrial taxa (e.g., *Elliptochloris*, Trebouxiophyceae), indicating a shift from “*Sphagnum* community” to a “terrestrial community”. Furthermore, we showed a decrease in the relative abundance of osmotrophs, including Fungi and parasites, including Oomycota (Peronosporomycetes). Overall, our work provides insight into the composition of *Sphagnum*-associated micro-eukaryotic communities and their response to drought and warming. We also show the potential of these organisms as indicators of ongoing changes. Identifying indicators of climate change using HTS might be a useful tool to identify state of the ecosystem and the direction of changes.

##  Supplemental Information

10.7717/peerj.9821/supp-1 Supplemental Information 1 Shows environmental variables in experimental plots in field manipulative experiment in a Polish peatland “WTD_Treatment” indicates water table treatment (DRY, CON –control, WET). “Warming” indicates warming treatment: OTC –warmed treatment with Open Top Chamber, ambient –treatment with ambient temperature.

10.7717/peerj.9821/supp-2 Supplemental Information 2 Shows abundance of each Operational Taxonomic Unit (OTU) in experimental plots in field manipulative experiment in a Polish peatland Row names correspond to the row names in the File S1, where details of the environmental variables for each plot can be found. The most abundant OTU sequence with raw taxonomic assignation based on the PR2 database are in File S3. OTU codes (column names starting with X followed by the number) correspond to the row names in the File S3. “WTD_Treatment” indicates water table treatment of the plot (DRY, CON –control, WET). “Warming” indicates warming treatment: OTC –warmed treatment with Open Top Chamber, ambient –treatment with ambient temperature.

10.7717/peerj.9821/supp-3 Supplemental Information 3 The most abundant sequence of the SSU rRNA V9 region in each of the Operational Taxonomic Unit “Sequence_id” –OTU code corresponds to the column names in File S1 and to the OTU codes in the article. “Sequence” –the most abundant sequence in the given OTU. ”Percent_identity” –percentage of identity of the most abundant sequence in the given OTU with the most similar sequence in the database. “Tax_assignation” –Taxonomic assignation of the OTUs done by pairwise alignment of the dominant sequences of each OTU against a selection of dereplicated V9 regions from the PR2 database (Guillou et al., 2013). Annotation _X of lineages in the PR2 database is resulting from a failure to assign a taxonomic identity (see Guillou L et al. 2013 for details). Reference: Guillou L et al. 2013. The Protist Ribosomal Reference database (PR2): a catalog of unicellular eukaryote Small Sub-Unit rRNA sequences with curated taxonomy. Nucleic acids research 41:D597-D604.

10.7717/peerj.9821/supp-4 Supplemental Information 4 Water table depth (WTD) in different water table treatments across seasons Water table treatments are indicated by symbols: ‘■’ represents CON treatment; ‘▴’ represents DRY treatment and ‘▾’ represents WET treatment. Plot shows mean values and bars indicate standard errors.

10.7717/peerj.9821/supp-5 Supplemental Information 5 Rarefaction curves of micro-eukaryotic sequences obtained from illumina sequencing of eDNA from *Sphagnum* mosses sampled in a field manipulative experiment in a Polish peatland showing saturation of the sequencing effort (A) the sequencing effort in each sample; (B) the sequencing effort for the whole dataset.

10.7717/peerj.9821/supp-6 Supplemental Information 6 Slope of the rarefaction curves in different water table treatments, seasons and warming treatments at the sample size of 2410 Filled symbols indicate warming in open top chambers while empty symbols indicate ambient temperature. Water table treatments are indicated by symbols: ‘■’ = CON (control); ‘▴’ = DRY and ‘▾’ = WET. Plot shows mean values and bars indicate standard errors. No significant differences were observed between treatments (ANOVA, *p* = 0.6 and *p* = 0.9, for warming and water table treatments, respectively).

10.7717/peerj.9821/supp-7 Supplemental Information 7 Redundancy analysis (RDA) ordination diagram showing patterns of eukaryotic communities on *Sphagnum* shoots as assessed by OTUs in the different plots Season (spring, summer and autumn sampling campaigns) is specified as a conditioning variable (“covariable”) partialled out before analysis, water level and temperature treatments are used as a variable. Filled symbols indicate warming (OTC) and empty symbols indicate ambient temperature. Water table treatments are indicated by symbols: ‘■’ = CON (control); ‘▴’ = DRY and ‘▾’ = WET. Centroid of each water table treatment is connected with its members with lines. Percentages of variance explained are given for each axis.

10.7717/peerj.9821/supp-8 Supplemental Information 8 Relative abundance of all OTUs assigned to *Elliptochloris*, a predominantly terrestrial taxa The relative abundance (%) is number of all OTUs assigned to *Elliptochloris*, divided by the total number of all OTUs combined.

10.7717/peerj.9821/supp-9 Supplemental Information 9 Mean, maximum and minimum water table depth (in cm below surface) across seasons and water table treatments in a field manipulative experiment in a Polish peatland CON –control treatment.

10.7717/peerj.9821/supp-10 Supplemental Information 10 Mean, maximum and minimum growing degree day across seasons and warming treatments in a field manipulative experiment in a Polish peatland OTC –open top chamber treatment.

10.7717/peerj.9821/supp-11 Supplemental Information 11 Summary of linear mixed effect models of diversity indices

10.7717/peerj.9821/supp-12 Supplemental Information 12 Diversity indexes (Shannon diversity, Evenness, OTU richness, Rarefied OTU richness) in each analyzed sample in a field manipulative experiment in a Polish peatland

10.7717/peerj.9821/supp-13 Supplemental Information 13 Micro-eukaryote indicator OTUs and corresponding taxonomic assignation of all indicators identified in the IndVal analysis with each treatment as a separate group in the field manipulative experiment in a Polish peatland *P*-value indicates the probability to have a higher IndVal by chance.

10.7717/peerj.9821/supp-14 Supplemental Information 14 Micro-eukaryote indicator OTUs and corresponding taxonomic assignation of all indicators identified in the IndVal analysis with OTC vs ambient as separate groups in the field manipulative experiment in a Polish peatland *P*-value indicate the probability to have a higher IndVal by chance.

## Additional Information and Declarations

### Field Study Permissions

The following information was supplied relating to field study approvals (i.e., approving body and any reference numbers): Field experiments were approved by The Regional Directorate for Environmental Protection in Bydgoszcz (WPN.6205.70.2011.KLD).

### Data Availability

The following information was supplied regarding data availability: The raw data are available in the Supplemental Files.
