# Supplementary figures and images for: Assessing the responses of Sphagnum micro-eukaryotes to climate changes using high throughput sequencing
Source: PeerJ. 2020 Sep 18;8:e9821. doi: 10.7717/peerj.9821 (PMC7505061; doi:10.7717/peerj.9821)

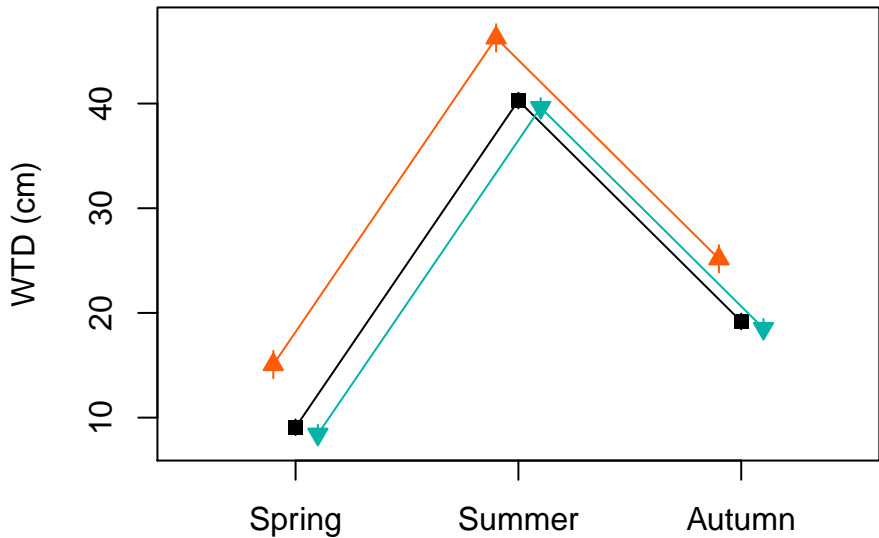

Supplement: Supplemental Information 4 — Water table treatments are indicated by symbols: ‘■’ represents CON treatment; ‘▴’ represents DRY treatment and ‘▾’ represents WET treatment. Plot shows mean values and bars indicate standard errors. [file peerj-08-9821-s004.pdf]

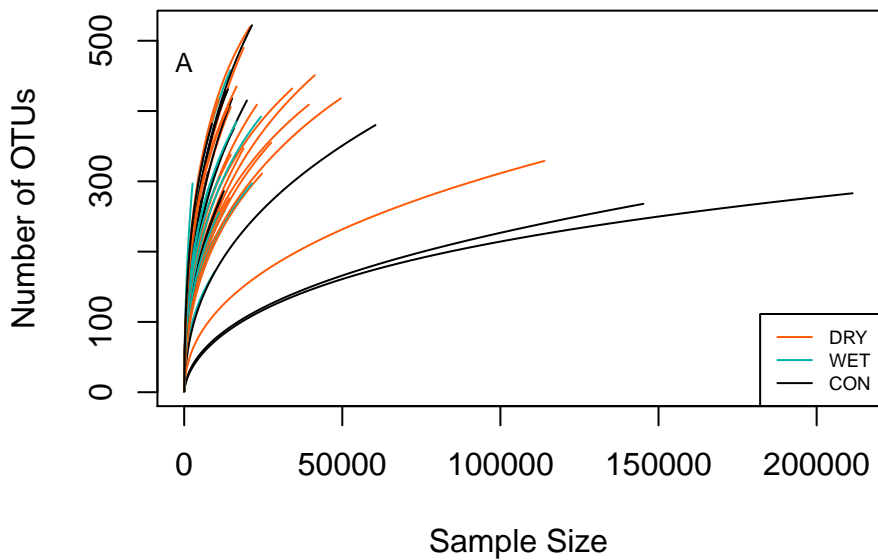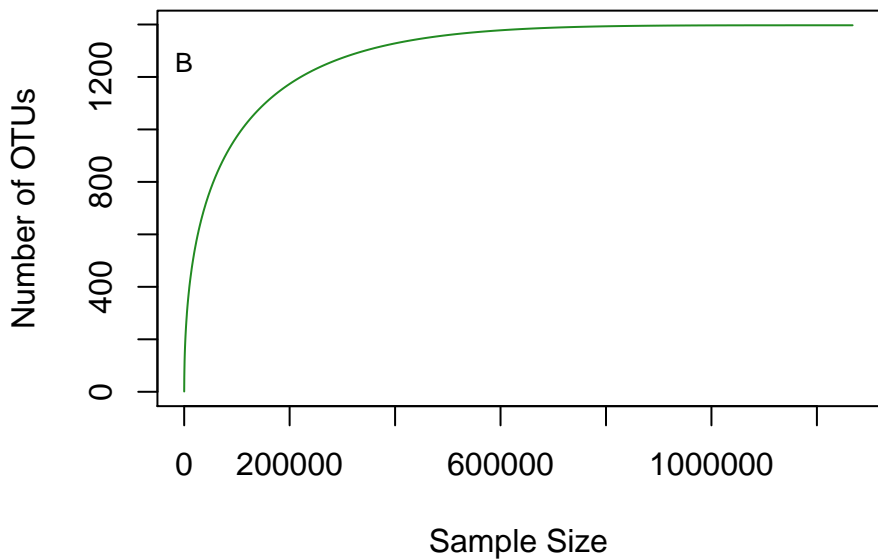

Supplement: Supplemental Information 5 — (A) the sequencing effort in each sample; (B) the sequencing effort for the whole dataset. [file peerj-08-9821-s005.pdf]

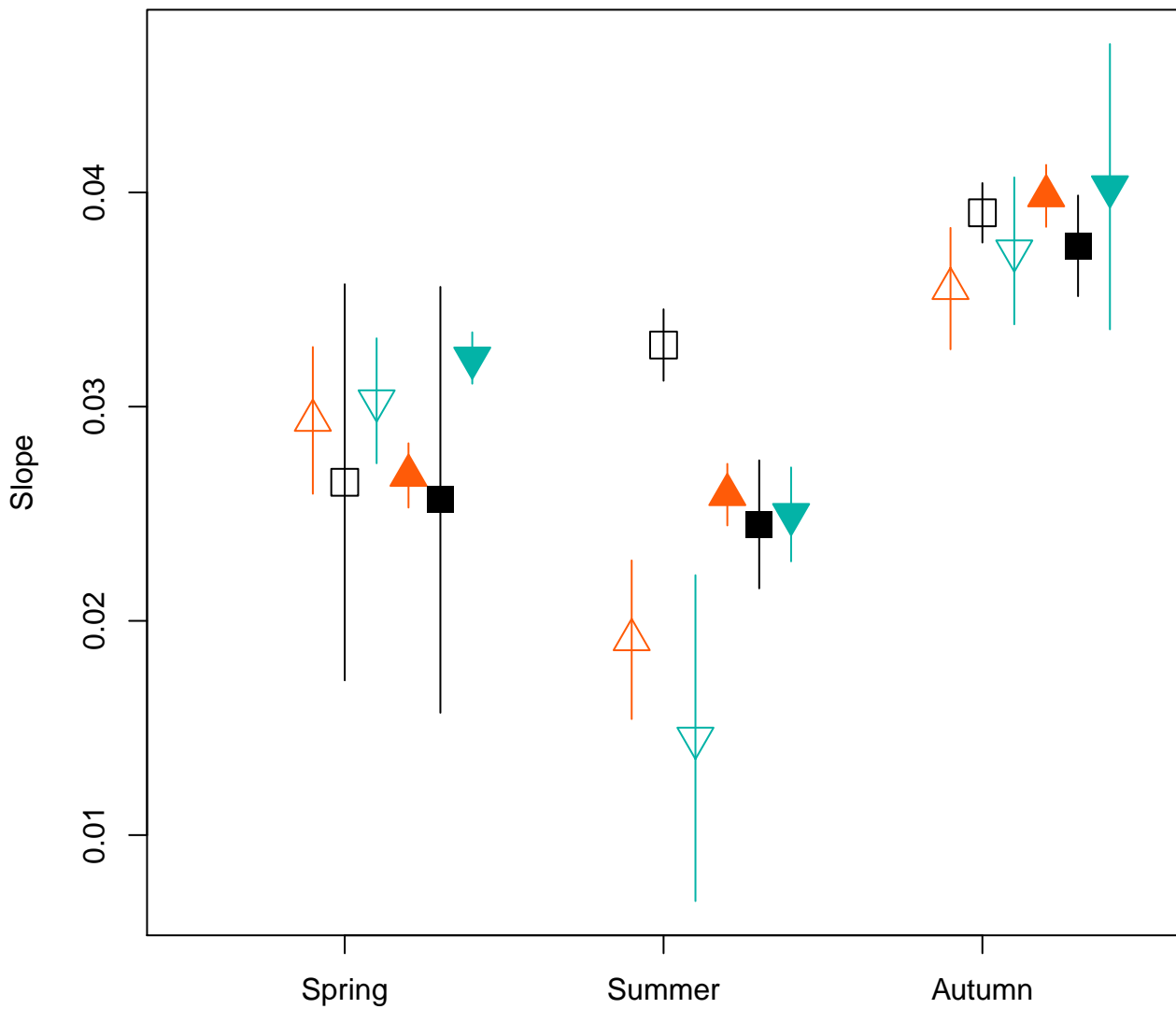

Supplement: Supplemental Information 6 — Filled symbols indicate warming in open top chambers while empty symbols indicate ambient temperature. Water table treatments are indicated by symbols: ‘■’ = CON (control); ‘▴’ = DRY and ‘▾’ = WET. Plot shows mean values and bars indicate standard errors. No significant differences were observed between treatments (ANOVA, p = 0.6 and p = 0.9, for warming and water table treatments, respectively). [file peerj-08-9821-s006.pdf]

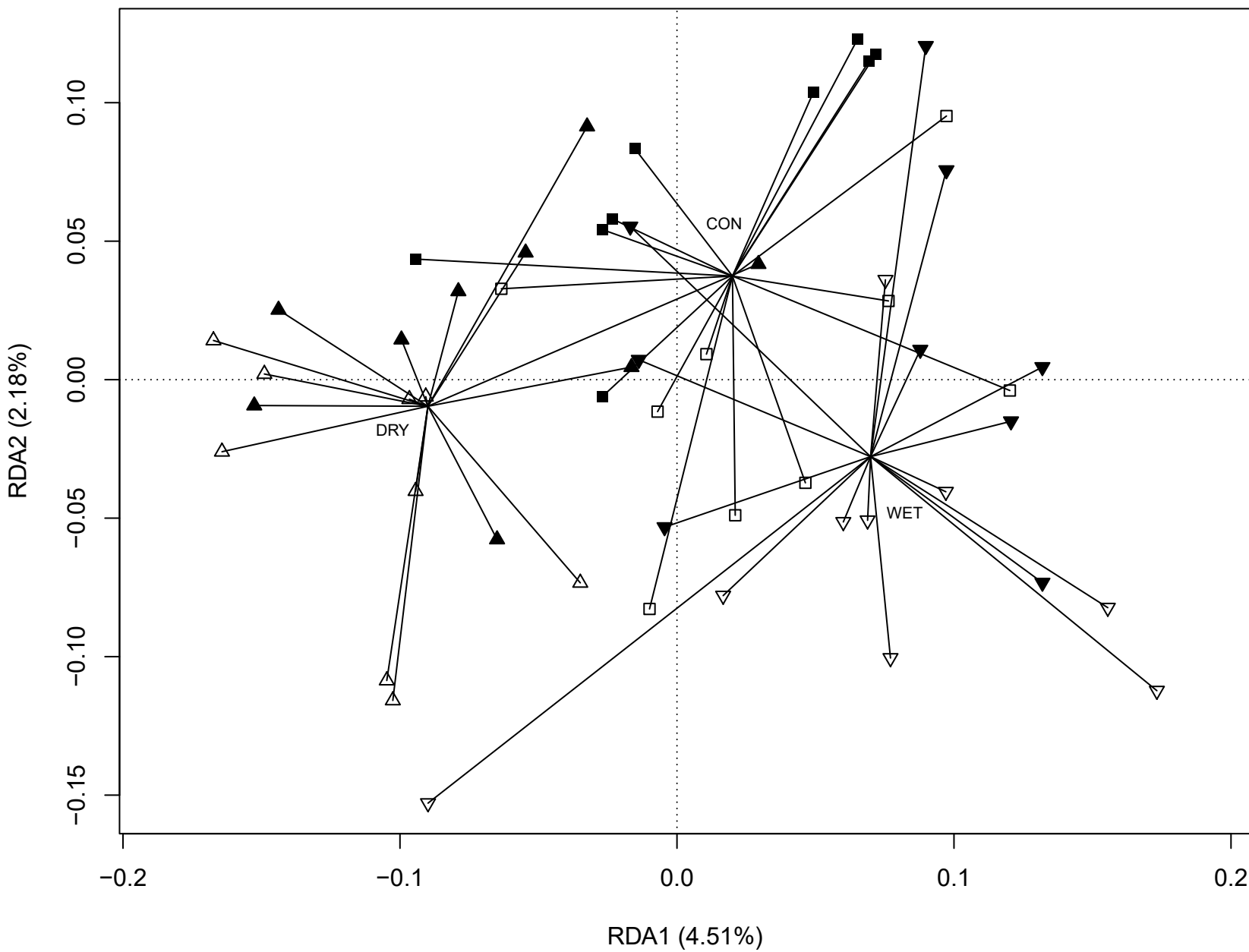

Supplement: Supplemental Information 7 — Season (spring, summer and autumn sampling campaigns) is specified as a conditioning variable (“covariable”) partialled out before analysis, water level and temperature treatments are used as a variable. Filled symbols indicate warming (OTC) and empty symbols indicate ambient temperature. Water table treatments are indicated by symbols: ‘■’ = CON (control); ‘▴’ = DRY and ‘▾’ = WET. Centroid of each water table treatment is connected with its members with lines. Percentages of variance explained are given for each axis. [file peerj-08-9821-s007.pdf]

# Elliptochloris

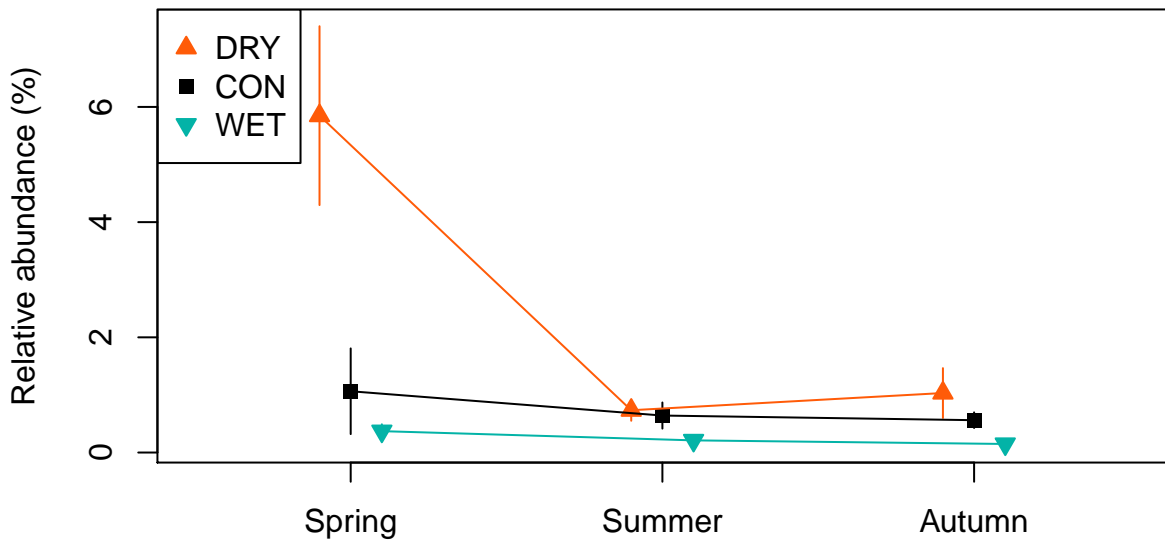

Supplement: Supplemental Information 8 — The relative abundance (%) is number of all OTUs assigned to Elliptochloris, divided by the total number of all OTUs combined. [file peerj-08-9821-s008.pdf]
